# Changes in the clinical presentation and outcomes of patients treated for severe malaria in a referral French university intensive care unit from 2004 to 2017

**DOI:** 10.1186/s13613-020-0634-4

**Published:** 2020-02-12

**Authors:** Jordane Lebut, Bruno Mourvillier, Nicolas Argy, Claire Dupuis, Camille Vinclair, Aguila Radjou, Etienne de Montmollin, Fabrice Sinnah, Juliette Patrier, Clément Le Bihan, Eric Magalahes, Roland Smonig, Eric Kendjo, Marc Thellier, Stéphane Ruckly, Lila Bouadma, Michel Wolff, Romain Sonneville, Sandrine Houzé, Jean-François Timsit

**Affiliations:** 1 AP-HP, Bichat Hospital, Medical and Infectious Diseases ICU (MI2), University of Paris, IAME, INSERM U1137 (IAME), 75018 Paris, France; 2 AP-HP, Bichat Hospital, Mycology Parasitology Department, Malaria National Reference Center, 75018 Paris, France; 3 University of Paris, IAME, INSERM, 75018 Paris, France; 4 Longjumeau Hospital, ICU, Longjumeau, France; 5 UMRS 1136, iPLESP, Institut Pierre-Louis d’épidémiologie et de santé publique, Sorbonne Université, 27, Rue Chaligny, 75571 Paris 12, France; 6 OUTCOMEREA Research Network, Drancy, France

**Keywords:** Severe malaria, Critically ill, Sepsis, Outcome, Artesunate

## Abstract

**Background:**

In France, the incidence of severe imported malaria cases increased since early 2000. Artesunate was available (temporarily use authorization) since mid-2011 in France and commonly used for severe malaria since early 2013. Thus, the study objectives were to describe the patients with severe imported malaria admitted in intensive care unit (ICU) and assess the changes in clinical presentation and outcomes before and after this date.

**Methods:**

Retrospective observational single-center study in the infectious diseases ICU of a referral university hospital, conducted on patients admitted for severe imported malaria from 2004 to 2017. Demographic variables, severity scores, WHO’s severity criteria on admission, treatment, and ICU and hospital lengths of stay were collected. Patients’ characteristics and outcomes were compared between both periods. A poor outcome was defined as the composite endpoint of death, or requirement for vasopressors, invasive mechanical ventilation and/or renal replacement therapy.

**Results:**

189 patients were included, 98 in 2004–2012 and 91 in 2013–2017, most often from West and Central African countries (96%). The number of WHO criteria for severe malaria was comparable in both groups, but SAPS II, SOFA and ICU length of stay were significantly higher in 2004–2012, while patients of African origin living in France were less frequent (*p* < 0.01). The outcome was poor for 41/98 cases in 2004–2012 and 12/91 cases in 2013–2017 (*p* < 0.01). The risk factors of poor outcome on the multivariate logistic regression were a neurological failure (adjusted odds ratio (adjOR = 3.23; 95% CI (1.03–10.08), *p* = 0.004), cardio-circulatory failure (adjOR = 9.92; 95% CI (2.34–42), *p* = <0.01) and creatinine blood levels > 265 µmol/L (adjOR = 10.76; 95% CI (3.17–36.53), *p* < 0.01). In the multivariate analysis, IV artesunate was not associated with a better outcome. Patients of African origin did not seem to have a better outcome than Caucasian patients or those from other origins (adjOR = 0.59; 95% CI (0.21–1.65), *p* = 0.31).

**Conclusion:**

Patients with imported malaria admitted in ICU in 2013–2017 were less severely ill than those in 2004–2012. These trends could be partially explained by the increasing proportion of African patients visiting friends or relatives or living in endemic areas.

## Background

Half of the world’s population is still exposed to malaria, causing an estimated 228 million of clinical cases associated to 405,000 deaths in 2018. The African continent is the most impacted by *Plasmodium* infection, with still 93% of all worldwide clinical malaria cases and 94% of worldwide deaths, mostly in young children. However, although the malaria incidence rate decreased by 21% between 2010 and 2015, thanks to successive health policies aiming to decrease *Plasmodium* spp. exposition in local populations of endemic countries, it remains stable since 2015 [1].

France is the non-endemic European countries most affected by malaria imported cases, increasing since 2012 [2–4], from 3580 estimated infected patients in 2012 to 5550 in 2018 (+ 55%). The associated mortality rate reached 0.39%. The increased incidence is likely explained by the growing number of African people visiting friends and relatives (VFR) in sub-Saharan countries and coming back to Western European countries such as France [5].

Importantly, during the last decade, the guidelines for the diagnosis and management of severe malaria cases have undergone major changes in endemic area and in France. Available in compassionate use in France since May 2011, artesunate became the first-line treatment for severe malaria. This drug is associated with a reduced mortality in endemic areas [6, 7], was shown to improve survival *versus* quinine in South-East Asia and Africa, to reduce parasite clearance time, and to shorten the ICU and hospital lengths of stay in non-endemic countries [8]. Its effectiveness and tolerance made it a good choice to replace intravenous quinine in European countries.

During the past decade, the characteristics of severe imported malaria cases referred to our intensive care unit (ICU) changed markedly. Patients were less often Caucasian tourists, presented less often with multiple organ failures, and seemed to recover more quickly. Therefore, we decided to further assess these epidemiologic changes and the potential impact of the widespread increase in the use of artesunate as a first-choice therapy on the improvement of prognosis.

The aim of this single-center study was to assess the evolution of the epidemiology, clinical presentation and outcome in severe imported malaria patients in an ICU of a referral hospital during the last 14 years. Our first objective was to assess over the years the clinical presentation, treatment and outcome in patients with severe imported malaria. The second one was to evaluate whether the changes in the outcome were correlated with the epidemiological evolution of patients with severe imported malaria, or with the availability of IV artesunate.

## Methods

### Study population

The study was performed in the infectious diseases ICU of Bichat–Claude Bernard University hospital in Paris. Every patient admitted with a diagnosis of severe malaria between January 2004 and December 2017 was included. Hospitalization report database and biological data were retrospectively collected.

According to the French recommendations for the management of severe imported malaria, adapted from the 2014 WHO’s definition [9], severe imported malaria cases were defined by the presence of *Plasmodium falciparum* parasites in peripheral blood (or, more rarely, of one of the four other *Plasmodium* species), associated with one or more defined severe clinical conditions or biological findings. Clinical criteria included neurological failure (with obnubilation, confusion or prostration, Glasgow Coma Scale < 11, or multiple seizures), respiratory failure (requirement for mechanical ventilation with PaO_2_/FiO_2_ < 300 mmHg or spontaneous breathing with PaO_2_ < 60 mmHg and/or respiratory rate > 30 per min), cardio-circulatory failure (systolic blood pressure < 80 mmHg despite adequate volume repletion or need for vasoactive drugs), hemorrhage, or jaundice. Laboratory criteria included hyperlactatemia (serum lactate > 2 mmol/L), acidosis (pH < 7.35), renal impairment (serum creatinine > 265 µmol/L), hyperparasitemia (> 4%), hypoglycemia (blood glucose < 2.2 mmol/L) and severe anemia (hemoglobin < 7 g/dL). Severity criteria were collected within the first hour of ICU admission.

### Data collection

Patients’ characteristics and outcomes were compared across two periods, namely from 2004 to end 2012 (1st study period) and from 2013 to 2017 (2nd study period), i.e., before and after artesunate became the first-choice treatment.

The demographic variables such as age, sex, ethnicity (Caucasian, African living in France or in Africa), visited endemic area, length of stay in endemic areas, chemoprophylaxis strategy applied, cause of travel, time between symptoms onset and ICU admission, previous therapy before ICU admission, medical history of prior malaria infection clinical and biological parameters during the first hour following ICU admission, curative treatment used, and ICU and hospital lengths of stay were collected. Malaria serology was also performed for all patients whom diagnosis was confirmed in our laboratory. As the exact time of the anti-malarial therapy start before ICU admission was not always available, it was recorded as a Likert scale variable (never, < 8 h, < 24 h, ≥ 24 h). The illness severity at ICU admission was assessed using the Simplified Acute Physiology Score II (SAPS II) and Sepsis-related Organ Failure Assessment (SOFA) score. A post-artesunate delayed hemolysis was sought in follow-up consultations and defined as a decrease in hemoglobin associated with haptoglobin < 0.1 g/L or LDH > 390 IL/L more than 7 days after treatment initiation with artesunate.

### Outcomes

We defined a poor outcome as a composite endpoint comprising death, or requirement for vasopressors, invasive mechanical ventilation (MV) and/or renal replacement therapy (RRT).

### Indirect immunofluorescence assay (IIFA)

The detection and the quantification of total antibodies against *Plasmodium falciparum* were used to identify a previous exposure to *Plasmodium* spp. Anti-plasmodial IgG/A/M antibodies were detected and quantified by serological screening based on indirect immunofluorescence assay (IIFA) using whole schizonts of the 3D7 *P. falciparum* strain as crude antigens, and fluorescein-linked anti-human IgG/A/M (Biorad^®^; Hercules, California, USA) as conjugate. Quantification of plasmatic antibody concentration was estimated by serologic titers. For statistical analysis, antibody titers were classified into three groups: negative if < 1:64; positive for titers from 1:64 to 1:1024 and highly positive for titers > 1:1024.

### Statistical analysis

Univariate analysis (Mann–Whitney or Chi-square tests as appropriate) was performed to unveil differences between both study periods. A similar analysis was used to select variables associated with poor outcome. Quantitative clinical and biological variables were transformed into dummy variables according to WHO criteria for severity. Variables with a *p* value of 0.1 or less were proposed for selection in a logistic regression model with stepwise selection stratified by period. Data are presented as median (interquartile range) or numbers (%). We used the SAS 9.4 software for all statistical analyses.

## Results

### Patient characteristics

**Fig. 1:**
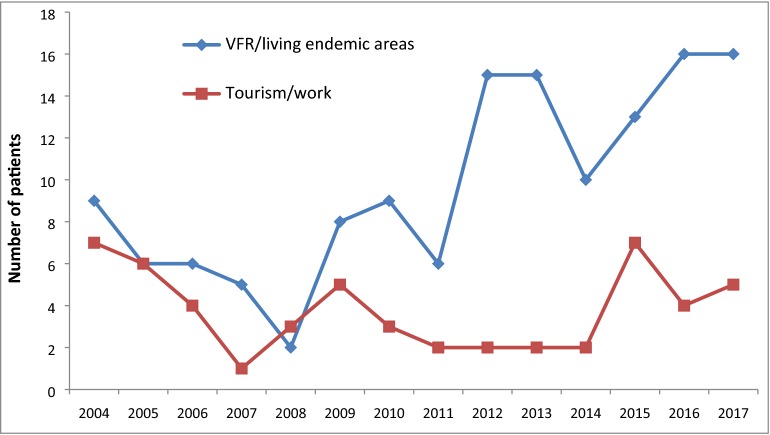
Evolution of the number of severe imported malaria cases according to the reasons for stay in endemic areas. VFR: visiting friends and relatives

From 2004 to 2017, 189 patients were admitted to our ICU for severe imported malaria. Demographic data are shown in Table 1. The median age was 45.3 years and 63% of the patients were male. The infection was most often acquired in West and Central Africa (96%). Most patients travelled to visit friends or relatives (52%) and took a partial anti-malarial chemoprophylaxis (94%). Thirty-seven patients (19%) were living in endemic countries and were travelling in France. The proportion of European people travelling for tourism or work was below one-third and seemed to be decreasing over the years (Fig. 1). Among travellers, the median duration of stay in endemic area was 30 days. The median duration of symptoms before ICU admission was 6 days.

**Table 1:** Characteristics of patients with severe imported malaria

Characteristics	Total	2004–2012	2013–2017	p value
Number of patients	189*	98	91	
Gender, male	119 (63)	65 (66)	54 (59)	0.32
Age, median in years [IQR]	45 [31; 56]	45 [29; 56]	45 [33; 57]	0.33
Clinical history of previous malaria	44 (23)	16 (16)	28 (31)	0.02
Positive malaria serology	89 (47)	45 (66)	44 (63)	0.68
Highly positive (IIFA > 1:1024)	29 (15)	17 (17)	12 (13)	0.36
Positive (IIFA from 1:64 to 1:1024)	60 (32)	28 (29)	32 (35)	.
Negative (IIFA < 1:64)	49 (26)	23 (23)	26 (29)	.
Missing	51 (27)	30 (31)	21 (23)	.
Ethnic group				< .01
Caucasian	51 (27)	36 (37)	15 (16)	
African	138 (73)	62 (63)	76 (84)	
*African living in France*	*121 (64)*	*53 (54)*	*68 (75)*	.
*African living in Africa*	*17 (9)*	*9 (9)*	*8 (9)*	.
Cause of travel				0.07
Visiting friends and relatives/living in endemic areas	136 (72)	65 (66)	71 (78)	
Europeans travelling for tourism or work	53 (28)	33 (34)	20 (22)	.
Complete anti-malarial chemoprophylaxis	12 (6)	6 (6)	6 (7)	0.89
Travel in West and Central Africa	182 (96)	92 (94)	90 (99)	0.07
*Plasmodium falciparum*	184 (97)	95 (97)	89 (98)	0.71
Time from symptoms onset to ICU admission, median in days [IQR]	6 [4, 9]	6 [4, 9]	5 [3, 9]	0.08
Treatment started at the time of ICU admission	91 (48.9)	47 (49.5)	44 (48.4)	0.43
Treatment started < 8 h before ICU admission	59 (31.7)	27 (28.4)	32 (35.2)	
Treatment started < 24 h before ICU admission	11 (5.9)	8 (8.4)	3 (3.3)	
Treatment started > 24 h before ICU admission	25 (13.4)	13 (13.7)	12 (13.2)	
ICU length of stay, median [IQR]	2 [2, 4]	4 [2, 7]	2 [2, 3]	< .01
Hospital length of stay, median [IQR]	7 [5, 13]	8 [6, 15]	7 [5, 10]	0.05
SAPS II, median [IQR]	25 [18; 36]	28 [20; 36]	22 [16; 35]	0.03
SOFA, median [IQR]	6 [4, 8]	6 [5, 8]	5 [4, 7]	< .01
Mortality	7 (4)	5 (5)	2 (2)	0.29

ICU: intensive care unit; IQR: interquartile range; IIFA: indirect immunofluorescence assay; SAPS: Simplified Acute Physiology Score; SOFA: Sequential Organ Failure Assessment

* Except for malaria serology there was no missing data of the independent variables

*Plasmodium falciparum* was identified in 184 cases out of the 189 patients (97%). The other species responsible for severe malaria were *Plasmodium vivax* (three patients), *Plasmodium ovale* (one patient) and *Plasmodium malariae* (one patient). These latter patients experienced more specifically circulatory failure (four patients) and respiratory distress syndrome (one patient).

The median parasitemia was 5.3% [1.5;9] and hyperparasitemia above 4% was the most commonly observed criteria of severe malaria (59%), as well as hyperlactatemia above 2 mmol/L (41%), jaundice or bilirubin blood levels above 50 µmol/L (39%). Although neurological failures were often observed (36%), only one out of ten patients presented a Glasgow Coma score below 11 or multiples seizures.

Underlying chronic conditions were reported in 31% of patients (58/189), most often hypertension and diabetes mellitus. The HIV status was known positive in 10% (18/189), two of them were at AIDS stage. Two patients were 4 months pregnant.

### First and second study periods

The proportion of people living in endemic areas or travelling for VFR increased from 66% in the first period to 78% in the second period (*p* = 0.07). During the second period, the number of severe imported malaria cases in Caucasian people decreased, while that in African people living in France increased significantly (*p* < 0.01). A medical history of prior malaria was less frequently observed during the first period than after, with, respectively, 16 (16%) and 28 (31%) patients (*p* = 0.02), although the proportions of positive malaria serology were comparable between periods. The lack of observance of anti-malarial chemoprophylaxis was deemed similar across periods, with still less than one patient out of ten taking a complete chemoprophylaxis. The platelet count was not different between periods (2004–2013: 44 G/L [26; 63.5]; 2013–2017: 51 G/L [30; 84]; *p* = 0.08).

Except for renal insufficiency (30% before 2013, 18% after 2013, *p* = 0.05), the illness severity criteria were comparable between both periods (Table 2), including the median parasitemia. Even if the number of WHO criteria for severe malaria on admission was similar between both groups (median = 2, *p* = 0.28), the ICU severity scores were significantly lower during the second period: the median SAPS II decreased from 28 to 22 (*p* = 0.03) and the median SOFA score from 6 to 5 (*p* < 0.01). During the second period, the median ICU length of stay was shorter by 2 days (*p* < 0.01) and the median hospital length of stay by 1 day (*p* = 0.05).

**Table 2:** Criteria leading to classification as severe malaria on admission

Severe malaria criteria	Total	2004–2012	2013-2017	*p* value
Neurological failure: obnubilation, confusion, GCS < 11 or multiple seizures	68 (36)	35 (36)	33 (36)	0.94
Glasgow Coma Scale < 11	17 (9)	12 (12)	5 (5)	0.13
Multiples seizures	8 (4)	6 (6)	2 (2)	0.18
Respiratory failure	11 (6)	5 (5)	6 (7)	0.66
Cardio-circulatory failure	36 (19)	16 (16)	20 (22)	0.31
Hemorrhage	3 (2)	2 (2)	1 (1)	0.6
Clinical jaundice or bilirubin > 50 µmol/L	73 (39)	42 (43)	31 (34)	0.21
Hyperlactatemia > 2 mmol/L	77 (41)	42 (43)	35 (38)	0.54
Lactate, median [IQR]	2.1 [1.2; 3.2]	2.1 [1.2; 3.5]	1.8 [1.2; 2.8]	0.32
Acidosis: pH < 7.35	21 (11)	14 (14)	7 (8)	0.15
pH, median [IQR]	7.4 [7.4; 7.5]	7.4 [7.4; 7.5]	7.4 [7.4; 7.5]	0.2
Renal impairment: serum creatinine > 265 µmol/L	45 (24)	29 (30)	16 (18)	0.05
Creatinine, median [IQR]	120 [82; 247]	140 [92; 280]	107 [77; 197]	< .01
Hyperparasitemia > 4%	112 (59)	59 (60)	53 (58)	0.78
Parasitemia, median in % [IQR]	5.3 [1.5; 9]	5.3 [2, 10]	5.3 [1.2; 7.7]	0.3
Hypoglycemia: blood glucose < 2.2 mmol/L	1 (0.5))	1 (1)	0 (0)	–
Severe anemia: hemoglobin < 7 g/dL	22 (12)	15 (15)	7 (8)	0.1
Number of severe criteria, median [IQR]	2 [1, 4]	2 [1, 4]	2 [1, 3]	0.28

GCS: Glasgow Coma Scale; IQR: interquartile range

The proportion of patients treated with artesunate was 12.2% (12/98 patients) during the first period as artesunate became progressively available in France since May 2011. After 2012, it reached 92.3% (84/91 patients) while the other seven patients received intravenous quinine, including four cases treated in another hospital before ICU admission, the two pregnant women and the case of *Plasmodium malariae*. The anti-malarial treatment was started before ICU admission in one patient out of two, without any significant difference between both periods (*p* = 0.43).

### Outcome

**Fig. 2:**
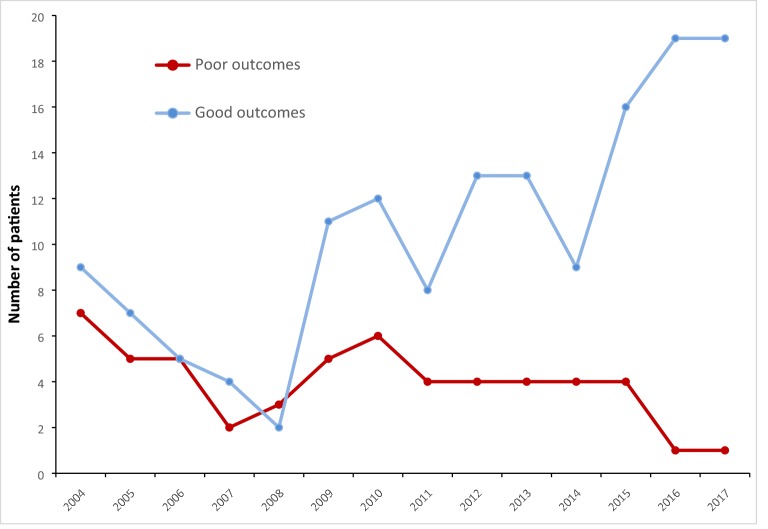
Evolution of the number of poor outcomes and good outcomes of severe imported malaria cases

A poor outcome, comprising death, requirement for vasopressors, invasive mechanical ventilation and/or renal replacement therapy, occurred in 41 (42%) and 12 (13%) patients during each period, respectively (*p* < 0.01) (Table 3). Patients’ characteristics and severity criteria at ICU admission, according to outcome are shown in Table 4. The evolution of the number of poor and good outcomes over time is shown in Fig. 2.

**Table 3:** Outcome of patients with severe malaria

Outcome	All patients	2004–2012	2013–2017	*p* value
Death	7 (4)	5 (5)	2 (2)	0.29
Need of vasopressors	19 (10)	13 (13)	6 (7)	0.13
Need of mechanical ventilation	34 (18)	28 (29)	6 (7)	< .01
Need of renal replacement therapy	35 (19)	26 (27)	9 (10)	< .01
Poor outcome	53 (28)	41 (42)	12 (13)	< .01

**Table 4:** Patients’ characteristics and severity criteria at ICU admission, according to outcome

Patients characteristics	Good outcome	Poor outcome	OR	95% CI	*p* value
Number of patients	136	53			
Gender, male	85 (62)	34 (64)	0.96	[0.48; 1.92]	0.91
Age, median [IQR]	45 [31; 57]	48 [31; 54]	1.01	[0.98; 1.02]	0.83
Clinical history of prior malaria	39 (29)	5 (9)	0.32	[0.11; 0.88]	0.03
Positive malaria serology	68 (64)	21 (66)	1.02	[0.44; 2.38]	0.96
Ethnic group					
Caucasian	27 (20)	24 (45)	1		0.03
African	109 (80)	29 (54)	0.3	[0.15; 0.6]	< .01
African living in France	97 (71)	24 (45)	0.37	[0.17; 0.76]	< .01
African living in Africa	12 (9)	5 (9)	0.56	[0.16; 1.92]	0.36
Cause of travel					
Visiting friends and relatives/living in endemic areas	106 (78)	30 (57)	0.42	[0.2; 0.85]	0.02
Europeans travelling for tourism or work	30 (22)	23 (43)	1		
Complete chemoprophylaxis	11 (8)	1 (2)	0.2	[0.02; 1.67]	0.14
Time from symptoms onset to ICU admission, median in days [IQR]	5 [4, 8]	7 [4, 9]	1.04	[1.01; 1.19]	0.02
Treatment started before ICU admission	67 (49)	31 (58)	1.49	[0.76; 2.94]	0.24
Treatment administered					
Artesunate	81 (60)	15 (28)	0.72	[0.25; 2.1]	0.55
Intravenous quinine	55 (40)	38 (72)	1		
SAPS II, median [IQR]	21 [16, 30]	41 [31; 56]	1.14	[1.09; 1.19]	< .01
SOFA, median [IQR]	5 [3, 6]	8 [7, 12]	1.92	[1.55; 2.37]	< .01
Parasitemia median in % [IQR]	5.1 [1.6; 8.0]	5.9 [1.5; 13.6]	1.03	[0.99; 1.07]	0.16

Severity criteria	Good outcome	Poor outcome	OR	95% CI	*p* value
Neurological failure	38 (28)	30 (57)	3.98	[1.94; 8.15]	< .01
Respiratory failure	2 (1)	9 (17)	23.61	[4.38; 127]	< .01
Cardio-circulatory failure	17 (12)	19 (36)	10.62	[3.67; 30.68]	< .01
Bilirubin > 50 µmol/L	49 (36)	24 (45)	1.32	[0.67; 2.6]	0.42
Serum creatinine > 265 µmol/L	16 (12)	29 (55)	8.86	[3.98; 19.71]	< .01
Severe anemia: hemoglobin < 7 g/dL	15 (11)	7 (13)	0.95	[0.35; 2.59]	0.92
Acidosis: pH < 7.35	5 (4)	16 (30)	11.53	[3.71; 35.83]	< .01
Hyperlactatemia > 2 mmol/L	46 (34)	31 (59)	2.86	[1.44; 5.69]	< .01
Hyperparasitemia > 4%	78 (57)	34 (64)	1.33	[0.67; 2.64]	0.42
Number of severity criteria, median [IQR]	2 [1, 3]	4 [2, 5]	2.16	[1.66; 2.81]	< .01

ICU: intensive care unit; IQR: interquartile range; SAPS: Simplified Acute Physiology Score; SOFA: Sequential Organ Failure Assessment; NB: for quantitative variable OR value is given per point of increase of the variable

Seven patients died during hospital stay (3.7%). Five of them died within the first week of care, and the two latter after a prolonged hospitalization stay and because of severe comorbidities. Four of these seven patients were transferred from another hospital and three of them did not receive any anti-malarial treatment before admission in our ICU. During their ICU stay, 19 patients (10%) needed vasopressors. An invasive mechanical ventilation was initiated in 35 (19%) patients, for 5 days in median. Twenty-three patients were intubated because of neurologic impairment, ten for respiratory distress, one for hemorrhagic shock and one for cardiac arrest. Thirty-six patients (19%) needed a renal replacement therapy throughout their full stay in ICU, for 4 days in median. Only two patients still needed hemodialysis after 1 month of care; however, their renal function recovered after 3 and 6 months, respectively. Erythrocytes transfusions were reported in 17 patients (9%) during ICU stay. Two patients suffered from splenic rupture. A post-artesunate delayed hemolysis was reported in follow-up consultations with ID physicians for 8/84 (9.5%) patients of the 2nd period. Symptomatic hypoglycemia occurred in three ICU patients, all treated with intravenous quinine. Only one patient presented severe hypoglycemia on admission before quinine administration.

In univariate analysis, the risk factors of poor outcome were neurological failure, respiratory failure, circulatory collapse, and creatinine serum level above 265 µmol/L.

In multivariate analysis, the risk factors of poor outcome were neurological failure, circulatory collapse and creatinine serum level above 265 µmol/L (Table 5). African-origin patients did not seem to have a better outcome than Caucasian or other origin patients (adjOR = 0.59; 95% CI (0.21–1.65), *p* = 0.31). When forced in the final prognostic model, artesunate-based therapy did not appear to be different from quinine-based therapy in term of prognosis (adjOR = 0.29; 95% CI (0.04–2.03), *p* = 0.21).

**Table 5:** Independent predictors of poor outcome at ICU admission, multivariate analysis

Patients characteristics	Estimate	Std error	adjOR	95% CI	*p* value
Ethnic group					0.31
Caucasian and others	Ref		1		
African	− 0.53	0.53	0.59	[0.21; 1.65]	
Treatment administered					0.21
Artesunate	Ref		1		
Intravenous quinine	1.23	0.99	3.43	[0.49; 23.9]	
Severity criteria on admission					
Neurological failure	1.17	0.58	3.23	[1.03; 10.08]	0.04
Cardio-circulatory failure	2.3	0.74	9.92	[2.34; 42]	< .01
Serum creatinine > 265 µmol/L	2.38	0.62	10.76	[3.17; 36.56]	< .01

Std error: standard error; adjOR: adjusted odds ratio; 95% CI: 95% confidence interval

NB: variables entered in the stepwise logistic regression model at the first step were: ethnic group; parasitemia greater than 4%, neurological failure; shock; lactate level greater than 1_8 mmol/L; pH < 7.35; serum creatinine > 265 µmol/L; bilirubin level > 50 IU; clinical history of prior malaria; cause of travel

## Discussion

The major finding of our study conducted in a referral ICU for severe imported malaria in the Parisian area is that, despite no clear modification of the number and types of WHO criteria of patients throughout both study periods, the patients had a less severe disease as estimated by the SAPS II and a better prognosis during the 2013-2017 time period. These changes in presentation and prognosis were not related to an earlier initiation of therapy and not clearly related to the widespread use of artesunate, but were associated with changes in the population over time. Patients were hospitalized with a significantly lower SAPS II scores, but without significant modifications of the number of severity criteria at ICU admission.

Among 189 patients admitted in this ICU, the in-hospital mortality was 3.7%, lower than that described in previous studies among patients admitted for severe imported malaria [10–12]. In the multicentric SIMA study [10], 35 patients (including two deaths) of our ICU were included with a lower mortality rate than in other centers (5.7% vs 10.9%). These results might be explained by the expertise of our ICU in the management of severe imported malaria. Recently, the French Artesunate Working Group analyzed the cohort of all severe imported malaria patients reported from 2011 to 2017, treated with artesunate or quinine, and observed a death rate of 3.2%, comparable to our result [13].

As the mortality associated with severe imported malaria is low, we defined a poor outcome as the composite endpoint of death, need of vasopressors, mechanical ventilation and/or renal replacement therapy. The association between severity criteria of malaria such as serum creatinine level above 265 µmol/L or cardio-circulatory, respiratory or neurological failures and our poor outcome seems obvious, but these criteria are universal predictors of poor prognosis on ICU admission. Acidosis and hyperlactatemia were also associated with a poorer prognosis. Other severity criteria of malaria, such as hyperparasitemia above 4%, did not seem associated with a poorer prognosis. Importantly, patients of African origin living in France, patients with prior exposure to malaria and with a shorter time from onset symptoms to ICU admission, had a better prognosis.

One of the explanations for the rise of severe imported malaria cases for the French national reference center was a significant and regular decrease in the proportion of people taking complete chemoprophylaxis [2]. We did not observe such a drop in our population over time, as our rate of patients who took complete anti-malarial chemoprophylaxis remained steadily very low. This shows that an important work on the education of these patients is still needed.

On the other hand, the French Reference Center explained the decreasing mortality rate in the recent years by the preponderant part of African-origin patients in severe imported malaria cases having a self-reported previous history of malaria [2]. A prior exposure to *Plasmodium falciparum* seems to induce a protection against severe malaria by generating antibody levels which are able to significantly reduce the circulating and sequestered parasite burden [13]. A persistence of some kind of protection after leaving malaria endemic areas is suspected, but the number of years covered by that protection is not well defined and needs further studies. In our work, we also observed a better outcome for patients with prior exposure, but we did not identify any correlation between a positive malaria serology and a good outcome. Data were missing for 51 patients (27%), mostly because those patients were transferred from another hospital. This finding questioned the need to develop better immunological tools to identify pre-exposed patients. While the number of travellers remained stable over the years, the proportion of patients living in endemic areas or VFR increased since 2008 (Fig. 1). Numerous associations between genetic polymorphisms and prognosis of severe malaria have been advocated [14–20], but formal confirmations using large multicenter worldwide cohorts are lacking. Independently of definite pathophysiological explanation, the changes in population may be one of the reasons for the better outcome of patients with severe imported malaria during the second period.

We observed several cases of African patients living in Africa hospitalized for severe imported malaria in our hospital. This population of African people travelling in France to visit friends and relatives was limited, but it exemplified that African adults may present severe malaria which needs ICU management in European countries. The worldwide decreased exposure to *Plasmodium* species with the rise of preventive measures induced a decreased incidence of malaria cases [21]. Perraut et al. found that immunity with antigen-specific antibody declined in the youngest population in endemic areas. The consequence of this waning immunity might be an increased incidence of cases of severe imported malaria in the future [22].

The development of new diagnostic tools as well as new recommendations for malaria management such as the introduction of artesunate as the first-line of treatment for severe imported malaria since 2013 could also have had an impact on the mortality rate of severe imported malaria. However, no difference were observed in the time interval between symptoms onset to ICU admission across both periods. SIMA and PALUREA, the two largest studies of imported malaria in France [10, 11], were conducted before the availability of artesunate. Their mortality rates were higher than in our study, with death occurring during the first week of hospitalization in three out of four patients. In these two studies, the proportion of patients needing the use of vasopressors, mechanical ventilation or renal replacement therapy is shown in table E1, as well as a review of other studies of severe imported malaria [8, 23, 24]. The risk factors associated with mortality in the SIMA cohort study were an older age, a low Glasgow Coma Scale score and a high parasitemia. In the PALUREA study, three host-related biomarkers were associated with severe malaria, namely a high level of procalcitonin and sTREM-1, and a low level of albumin. As parasitemia only reflects the circulating parasites, which are less pathogenic than the sequestered parasites, another parasite-related biomarker, PfHRP2, is usually considered more relevant than parasitemia to identify severe malaria [25]. However, this biomarker does not allow to differentiate severe malaria with organ injuries from a “simple” hyperparasitemic severe malaria case [13, 26]. All those biomarkers were not systematically collected in our retrospective study and could not be analyzed.

The TropNet severe malaria study showed that a treatment with artesunate reduced the parasite clearance time and was associated with shorter ICU and hospital lengths-of-stay [8]. The initiation of an anti-malarial treatment is an emergency and previous studies demonstrated that a better prognostic was associated with a shorter time between the first symptoms and the initiation of treatment administration [27, 28]. However, we could not demonstrate that patients with better outcome had an earlier initiation of treatment. In our study, even a treatment started before ICU admission was not significantly associated with a better outcome. Nevertheless, we observed that 3/7 deaths occurred in patients transferred from another hospital without any administration of anti-malarial agents. We did not analyze the anti-malarial treatment that may have followed the initial administration of artesunate or quinine, because we estimated that the outcome of severe malaria would depend of the first anti-malarial treatment.

The main limitation of our study is the retrospective and monocentric design in a referral French ICU specialized in infectious diseases. Mortality might have been biased by the fact that our ICU had a long experience in treating severe imported malaria. The absence of difference between artesunate and quinine treatment in this study might be explained by a large experience in IV quinine use and the use of up-to-date standard of ICU care. Adverse effects reported with quinine use were very rare with only three symptomatic episodes of hypoglycemia. The absence of difference may also be due to a lack of study power because of an insufficient number of patients. In 2012, a retrospective study in the United Kingdom compared 24 patients treated with artesunate to 167 patients treated with quinine [29]. The length of stay was shorter in the artesunate group. The authors mentioned that this improvement might have been due either to the artesunate use or to a change in the population admitted. Indeed, they observed a shift in the origins of the patients with a higher proportion of African patients in the artesunate group. We also observed a change in the profile of our patients over time, with a higher proportion of African patients visiting friends or relatives or living in endemic countries.

The French Artesunate Working Group has also yielded the absence of significant impact of artesunate use in France. Indeed, in a country with a high level of care, no difference was found in term of mortality rate or duration of stay of severe malaria treated with artesunate versus quinine [30]. However, artesunate is a safe and effective treatment of severe malaria that became a standard of care, even in high-income countries and our aim was not to question its superiority, but to identify other parameters responsible for the change in prognosis. Post-artesunate delayed hemolysis occurred in 9.5% of the patients, which seems very low compared to other studies [25]. This event was collected from follow-up consultation reports and may have been underestimated.

In our unit, we did not observe any treatment failure when using artesunate. Treatment failure was defined by the WHO as the inability to clear malarial parasitemia or prevent recrudescence after treatment. Factors identified by the WHO to contribute to treatment failure were poor patient compliance, drug interactions and resistance [26]. In our study, 96% of the patients with severe imported malaria were coming back from West and Central Africa and only four patients travelled in Asia where cases of artesunate resistance have been described. The oral treatment following intravenous artesunate was artemisin-based combination therapy in most patients. Compliance to the oral treatment was not assessed in our study, as patients were usually already discharged from our ICU at this time.

## Conclusion

In our ICU, the majority of patients admitted with severe imported malaria were of African origin and acquired their infection in West and Central Africa. On admission, those patients were less severe during the period of artesunate use than before. These trends could be partially explained by changes in the exposed population, with an increasing proportion of patients of African origin in the second period.

## Supplementary information

**Additional file 1.** Additional table.

## Data Availability

Raw data are available upon request and detail protocol for research only.

## Abbreviations

adjOR: Adjusted odds ratio
AIDS: Acquired immunodeficiency syndrome
FiO_2_: Fraction of inspired oxygen
ICU: Intensive care unit
IIFA: Indirect immunofluorescence assay
IQR: Interquartile range
IV: Intravenous
MV: Mechanical ventilation
OR: Odds ratio
PaO_2_: Partial pressure in oxygen
RRT: Renal replacement therapy
SAPS: Simplified Acute Physiology Score
SOFA: Sequential Organ Failure Assessment
VFR: Visiting friends and relatives
WHO: World Health Organization
95% CI: 95% confidence interval
