# Correction to: Changes in the clinical presentation and outcomes of patients treated for severe malaria in a referral French university intensive care unit from 2004 to 2017

**DOI:** 10.1186/s13613-020-0646-0

**Published:** 2020-03-03

**Authors:** Jordane Lebut, Bruno Mourvillier, Nicolas Argy, Claire Dupuis, Camille Vinclair, Aguila Radjou, Etienne de Montmollin, Fabrice Sinnah, Juliette Patrier, Clément Le Bihan, Eric Magalahes, Roland Smonig, Eric Kendjo, Marc Thellier, Stéphane Ruckly, Lila Bouadma, Michel Wolff, Romain Sonneville, Sandrine Houzé, Jean-François Timsit

**Affiliations:** 1 AP-HP, Bichat Hospital, Medical and Infectious Diseases ICU (MI2), University of Paris, IAME, INSERM U1137 (IAME), 75018 Paris, France; 2 AP-HP, Bichat Hospital, Mycology Parasitology Department, Malaria National Reference Center, 75018 Paris, France; 3 University of Paris, IAME, INSERM, 75018 Paris, France; 4 Longjumeau Hospital, ICU, Longjumeau, France; 5 UMRS 1136, iPLESP, Institut Pierre-Louis d’épidémiologie et de santé publique, Sorbonne Université, 27, Rue Chaligny, 75571 Paris 12, France; 6 OUTCOMEREA Research Network, Drancy, France

## Correction to: Ann Intensive Care (2020) 10:21 10.1186/s13613-020-0634-4

After publication of the original article [1], we were notified that family names have been exchanged with the first names for all authors. Below the name are tagged correctly:

- Given name: Jordane
- Family name: Lebut
- Given name: Bruno
- Family name: Mourvillier
- Given name: Nicolas
- Family name: Argy
- Given name: Claire
- Family name: Dupuis
- Given name: Camille
- Family name: Vinclair
- Given name: Aguila
- Family name: Radjou
- Given name: Etienne
- Family name: de Montmollin
- Given name: Fabrice
- Family name: Sinnah
- Given name: Juliette
- Family name: Patrier
- Given name: Clément
- Family name: Le Bihan
- Given name: Eric
- Family name: Magalahes
- Given name: Roland
- Family name: Smonig
- Given name: Eric
- Family name: Kendjo
- Given name: Marc
- Family name: Thellier
- Given name: Stéphane
- Family name: Ruckly
- Given name: Lila
- Family name: Bouadma
- Given name: Michel
- Family name: Wolff
- Given name: Romain
- Family name: Sonneville
- Given name: Sandrine
- Family name: Houzé
- Given name: Jean-François
- Family name: Timsit

The original article has been corrected as well.
