# Supplementary material for: Changes in the clinical presentation and outcomes of patients treated for severe malaria in a referral French university intensive care unit from 2004 to 2017
Source: Ann Intensive Care. 2020 Feb 12;10:21. doi: 10.1186/s13613-020-0634-4 (PMC7016155; doi:10.1186/s13613-020-0634-4)
Supplement: Supplementary file 1 — Additional file 1. Additional table. [file 13613_2020_634_MOESM1_ESM.docx]

**Additional file**

**Changes in the clinical presentation and outcomes of patients treated for severe malaria in a referral French university intensive care unit from 2004 to 2017.**

**Authors:**

Lebut Jordane^1,4^ MD, Mourvillier Bruno^1,5^ MD, Argy Nicolas^2^, Dupuis Claire^1,3^ MD, PhD, Vinclair Camille^1^ MD, Radjou Aguila^1^ MD, de Montmollin Etienne^1,3^ MD, Sinah Fabrice^1^ MD, Patrier Juliette^1^ MD, Le Bihan Clément^1,3^ MD, Magalahes Eric^1^ MD, Smonig Roland^1^ MD , Kendjo Eric^5^, Thellier Marc^5^ , Ruckly Stéphane^6^ MSc, Bouadma Lila^1,3^ MD, PhD, Wolff Michel^1,3^ MD, PhD, Sonneville Romain^1,3^ MD, PhD, Houzé Sandrine^2^, Timsit Jean-François^1,3,6^ MD, PhD

**Changes in the clinical presentation and outcomes of patients treated for severe malaria in a referral French intensive care unit in a university hospital from 2004 to 2017**

| Table E1. Severe malaria review |  |  |  |  |  |  |  |  |  |  |
| --- | --- | --- | --- | --- | --- | --- | --- | --- | --- | --- |
| **Study title** | **Year** | **Date** | **Nb of patients** | **Treatment** | **Mortality (%)** | **MV (%)** | **Vasopressors (%)** | **RRT (%)** | **ICU LOS (IQR)** | **Hospital LOS (IQR)** |
| Severe Imported Falciparum Malaria: a cohort study in 400 critically ill adults (10) | 2010 | 2000-2006 | 400 | Quinine | 42 (10) | 116 (29) | 109 (27) | 81 (20 | 5 (3-8) | 10 (7-17) |
| Treatment outcome of intravenous artesunate in patients with severe malaria in the Netherlands and Belgium (17) | 2012 | 2007-2010 | 55 | Artesunate | 2 (3.6) | 6 (9) | 6 (9) | 5 (7) | 2 | 4.5 |
| Severe imported falciparum malaria among adults requiring intensive care: a retrospective study at the hospital for tropical diseases, London (12) | 2013 | 1994-2010 | 124 | Q / AS | 5 (4) | 46 (37) | 43 (35) | 43 (35) | 10 (7-19) | . |
| Imported falciparum malaria in adults: host- and parasite related factors associated with severity, The French prospective multicenter PALUREA cohort study (11) | 2016 | 2006-2010 | 155 | Quinine | 8 (5.2) | 42 (27) | 39 (25) | 26 (17) | 7.1 (mean) | 13.3 (mean) |
| Severe plasmodium falciparum malaria in the intensive care unit: a 6-year experience in Milano, Italy (16) | 2017 | 2010-2015 | 12 | Quinine | 0 (0) | 5 (42) | 4 (33) | 4 (33) | 5 | 11.5 |
| Severe malaria in Europe: a 8-year multicenter observational study (24) | 2017 | 2006-2014 | 185 | Q / AS | 3 (1.6) | 18 (10) | 24 (13) | 20 (11) | 3 vs 2 | 7 vs 6 |
| Present study. | 2019 | 2004-2017 | 189 | Q/AS | 7 (3.7) | 34 (18) | 19 (10) | 35 (19) | 2 (2-4) | 7 (5-13) |

MV: invasive mechanical ventilation; RRT: Renal replacement therapy; Q / AS: quinine/artesunate without distinctions ([31](#_ENREF_31))
